# Total vs. Bioavailable: Determining a Better 25(OH)D Index in Association with Bone Density and Muscle Mass in Postmenopausal Women

**DOI:** 10.3390/metabo11010023

**Published:** 2020-12-31

**Authors:** Nurdiana Z. Abidin, Soma R. Mitra

**Affiliations:** 1 School of Biosciences, Faculty of Science and Engineering, University of Nottingham Malaysia, 43500 Semenyih, Selangor, Malaysia; soma.mitra@nottingham.edu.my; 2 Lifestyle Science Cluster, Advanced Medical and Dental Institute, Universiti Sains Malaysia, 13200 Kepala Batas, Pulau Pinang, Malaysia

**Keywords:** obesity, osteoporosis, sarcopenia, vitamin D, osteosarcopenic obesity

## Abstract

The concurrent presence of low bone density (osteopenia/osteoporosis) and low muscle mass (sarcopenia) in older adults has led to the recognition of “osteosarcopenia” (OS) as a singular entity. Vitamin D may play important role in the manifestation of OS, in terms of intake, absorption, and bioavailability. Evidence suggests that bioavailable 25(OH)D may be a better indicator of Vitamin D compared to total 25(OH)D due to its weak bind to albumin, increasing its ‘availability’. The aim of this study was to assess total and bioavailable 25(OH)D levels in postmenopausal women and to determine their associations to bone density and muscle mass. We assessed body composition, bone density, and 25(OH)D indices of multiethnic, postmenopausal Malaysian women. A significant and negative correlation was found between body fat % and each index of 25(OH)D. Both bioavailable and total 25(OH)D were positively correlated with serum calcium and negatively correlated with iPTH(intact parathyroid hormone). VDBP(Vitamin D binding protein) level was significantly correlated with bioavailable 25(OH)D level, but not with the total 25(OH)D level. Stepwise regression analysis revealed that bioavailable, but not total, 25(OH)D was significantly correlated to bone density and muscle mass, (where stronger correlation was found with bone density), suggesting its superiority. Nevertheless, the low effect size warrants further studies.

## 1. Introduction

Studies have found that bone and muscle (and also adipose tissue) are endocrine organs that secrete molecules to communicate and form a cross-talk network with each other, and are regulated through the same musculoskeletal pathways [1]. Aging has been found to be one of the factors that cause disturbance to the mesenchymal stem cells (MSC) lineage commitment (which are precursors for bone, muscle and adipose tissue), and subsequently leading to tissue impairments. The tissue impairments tend to lead to multitude of disorders, namely osteoporosis and sarcopenia. To date, there is emerging evidence showing a close link between these two conditions [2]. The interconnected pathways between muscle and bone have led to the hypothesis that the improvement of one may be beneficial to the other. Therefore, the scientific and medical communities are working hard on studying the interconnectedness between these conditions in order to aid in their prevention and treatment. Recently, it has been suggested that vitamin D, which is an essential nutrient required for optimal absorption of dietary calcium and phosphate, may play a role in the development of osteosarcopenia (OS), where an individual has simultaneous manifestation of low bone density (osteoporosis) and low muscle mass (sarcopenia) at the same time [2]. Therefore, it was hypothesised that increased vitamin D levels may lower the risk of developing the combined disorder.

To understand the effect of vitamin D on the combined disorder, it is important to understand the effect of vitamin D on each of its component. Hypovitaminosis D (low vitamin D levels) has been found to be prevalent in people with bone and muscle wasting. Prior studies have shown low vitamin D levels to have adverse effects on the absorption of calcium, bone remodeling, and consequently, bone density [3,4]. Further, low vitamin D levels have also been found to be associated with increased parathyroid hormone, which contributes to the catabolism of bones [5,6,7]. In the case of muscle mass, studies have shown a significant correlation between vitamin D receptor polymorphisms and reduced muscle mass and function in older adults [8], suggesting the role of vitamin D in sarcopenia [9]. Among the obese populations, the prevalence of hypovitaminosis D is unfortunately higher than among the people of normal weight. Due to the fat-solubility of the micronutrient, where fat essentially acts as a ‘sink’, vitamin D levels in obese individuals tend to be much lower. Therefore, obese individuals are generally more susceptible to developing musculoskeletal health disorders due to their low vitamin D levels. 

Currently, there are still some controversies regarding the exact role of vitamin D in musculoskeletal health. Studies on the effects of vitamin D on muscle or bone health have shown conflicting results, likely due to the heterogeneity between studies [10,11]. Moreover, due to the design of studies, causality is impossible to determine [12,13,14]. Therefore, various hypotheses emerged to explain the conflicting results, among which, include the free hormone hypothesis.

### Free Hormone Hypothesis: Total vs. Bioavailable 25(OH)D

Currently, ‘total’ 25-hydroxyvitamin D (25(OH)D) is considered to be an indicator of vitamin D status. This metabolite reflects the overall body storage of the immediate vitamin D precursor that is hydroxylated to active 1,25-dihydroxyvitamin D. In the body, after undergoing the first hydroxylation in the liver, ‘total’ serum 25(OH)D is bounded to vitamin D binding protein (VDBP) and Albumin (Alb), which then transported to the kidney and get converted to hormonally active 1,25-dihydroxyvitamin D (1,25(OH)_2_D) [15]. In the circulation, most of both 25(OH)D and 1,25(OH)_2_D are bounded to VDBP (~85–90%), approximately 10–15% to Alb, and less than 1% is considered as ‘free’ and unbounded to anything [16]. Since the affinity of 25(OH) D or 1,25(OH)_2_D to Alb is weaker than to VDBP, the loosely bound fraction and the ‘free’ fraction together make up the ‘bioavailable’ 25(OH)D [17]. Free hormone hypothesis posits that the core activity of a hormone may be due to the ‘bioavailable’ fraction and its passive diffusion across the membrane. In the case of vitamin D, the majority of the vitamin in the blood is bounded to VDBP, rendering it unavailable for passive diffusion. Emerging evidence is showing that bioavailable 25(OH)D (the fraction that’s not bound to VDBP), may be the better indicator of vitamin D status compared to the ‘total’ 25(OH)D (*aka* the free hormone hypothesis) [18,19]. 

Currently, there are contradicting findings on the correlation of total 25(OH)D with bone density and muscle mass. Studies suggest that bioavailable fraction of 25(OH)D may be a better indicator for vitamin D status than total 25(OH)D in relation to musculoskeletal health. For example, a study by Lowe et al. [20] which investigated the differences in vitamin D status in postmenopausal South Asian and Caucasian women in the UK and its relationship to parathyroid hormone (PTH) concentration, biochemical markers of bone turnover and bone quality, found that although the South Asian women had significantly lower total 25(OH)D concentrations and higher serum PTH than the Caucasian women, they were not associated with significantly higher markers of bone resorption, or reduced bone quality. These findings suggest that factors other than just the level of total 25(OH)D might play a role in bone quality and health, which, based on various studies [19,21], are likely to be the bioavailable fraction of 25(OH)D. Perhaps the measurement of bioavailable 25(OH)D may need to be incorporated into the routine assessment to improve the determination of vitamin D status in diverse populations. 

To the best of our knowledge, very few studies were designed to assess bioavailable vitamin D status and its correlation to musculoskeletal health in community-dwelling postmenopausal women. Therefore, the aim of the current study was to assess the status of total and bioavailable 25(OH)D in community-dwelling postmenopausal women and determined if the bioavailable fraction of 25(OH)D is the better biochemical indicator for vitamin D status than total 25(OH)D in relation to muscle mass and bone density. 

## 2. Results

Table 1 shows the characteristics and body composition measurement of participants. Median and interquartile range (IQR) of age, age at menopause, and years since menopause are 59.0(10.0) years, 50.0(6.0) years and 8.0(11.0) years, respectively. Categorization based on BMI shows that the prevalence of ‘Obesity’ (BMI ≥ 27.5 kg/m^2^) was 48.0% (*n* = 68). Alternatively, if categorization was based on waist circumference (WC) and body fat percent (BFP), the proportion for Obese/Overweight were 63.0% (*n* = 87) and 86.0% (*n* = 121), respectively.

According to the standard cut-off based on appendicular skeletal muscle mass index (appSMMI) ≤ 5.7 kg/m^2^ [25], the prevalence for sarcopenia was 31.2% (*n* = 44). Based on the standard cut-off for broadband ultrasonic attenuation (BUA < 54 dB/MHz) [26], the prevalence for osteopenic/osteoporosis was 18.0% (*n* = 25). The median and IQR of total, bioavailable and free 25(OH)D were 51.0(23.85) nmol/L, 6.2(3.2) nmol/L, and 15.7(7.4) pmol/L, respectively. The results for each blood parameter were all within the normal range for adults.

Background information such as education level, smoking habit, alcohol-drinking habit, physical activity level and co-morbidities were collected from postmenopausal women using a questionnaire (Table 2). The current study found that a majority of the participants were educated at the secondary school level or higher (77.0%), non-cigarette smoking (98.6%), and non-alcohol drinking (97.8%) individuals. Half of the cohort (50.4%) reported to be ‘inactive’ during the week while the other half reported to have some amount of physical activity (other than regular types of activity such as household chores) at least 10 min per day. Forty-three percent (43.2%) of participants reported to not have or ever have been diagnosed with any type of disorders listed in the questionnaire.

Table 3 describes the characteristics of people with different statuses of vitamin D; deficient, insufficient, and replete. The majority of participants in this cohort were in the ‘vitamin D replete’ category (51.7%, >50 nmol/L), followed by ‘vitamin D insufficient’ (40.8%, 30–50 nmol/L) and ‘vitamin D deficient’ (7.5%, <30 nmol/L).

Results shows that participants with lower levels of vitamin D (deficient and insufficient groups) had significantly higher iPTH, body fat percent (BFP) (*p*-value < 0.05), and lower free and bioavailable 25(OH)D (*p*-value < 0.001) compared to participants with higher levels of vitamin D (replete group). Additionally, participants in the ‘deficient’ group had significantly lower bone density (BUA) and blood serum calcium level compared to the group with higher levels of vitamin D (replete) (*p*-value < 0.05). There were no significant differences of variables between ‘deficient’ and ‘insufficient’ groups except for bioavailable and free 25(OH)D (*p*-value < 0.05).

With regard to functional performance, participants with lower levels of vitamin D (deficient and insufficient) were found to have significantly lower endurance (walk speed) compared to participants with higher levels of vitamin D (replete, *p*-value < 0.01). No significant differences, however, were found for lower extremity strength (sit-to-stand test) and balance between the groups. There were also no significant differences found for any of the muscle indices, handgrip strength (HGS) and VDBP between the groups.

**Figure 1:**
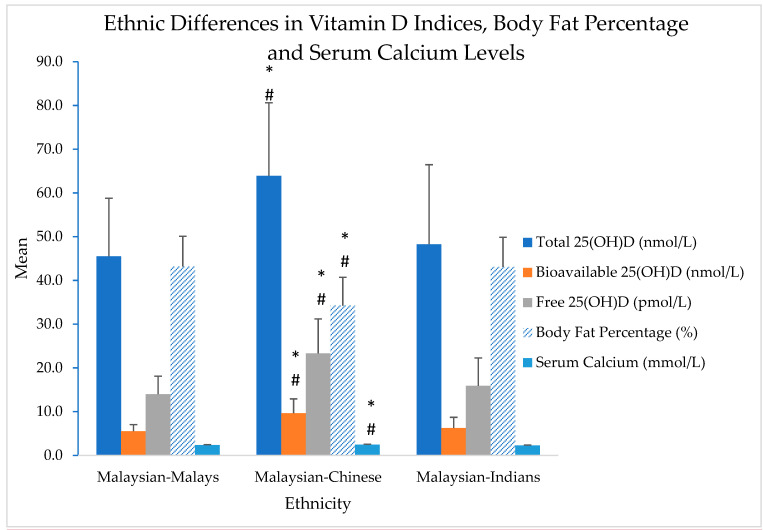
25(OH)D indices, body fat percentage and serum calcium levels according to ethnicity; Malaysian-Malays, *n* = 49, Malaysian-Chinese, *n* = 35, Malaysian-Indians = 36. Error bars: + 1 Standard deviation, * analysed using one-way ANOVA, with Tukey’s HSD Post-hoc test, # = different from Malaysian-Malays and Malaysian-Indians, *p*-value < 0.001.

Figure 1 shows ethnic differences between Malaysian-Malays, Malaysian-Chinese and Malaysian-Indians for 25(OH)D indices (total, free and bioavailable 25(OH)D), body fat percentage and serum calcium levels.

Results shows that Malaysian-Chinese had significantly higher levels of serum calcium, total, free and bioavailable 25(OH)D, and significantly lower body fat percentage compared to Malaysian-Malays and Malaysian-Indians (*p*-value < 0.05). Although there were slight variations in the levels of vitamin D indices between the Malaysian-Malays and Malaysian-Indians (Malaysian-Indians > Malaysian-Malays), no statistical significance was found. 

Table 4 shows the correlations of blood biomarkers with fat, bone, muscle, and functional performance. 

The current study found that each form of 25(OH)D (total and bioavailable) was negatively correlated with body fat percent (BFP) (*r* = −0.298, *r* = −0.380, respectively, *p*-value < 0.05) and positively correlated with bone density, BUA dB/MHz (*r* = 0.199, *r* = 0.234, respectively, *p*-value < 0.05). With regards to functional performance, gait speed (GS) was found to be positively correlated to total 25(OH)D (*r* = 0.191, *p*-value < 0.05), while lower extremity strength (STS) was found to be positively correlated with bioavailable 25(OH)D (*r* = 0.202, *p*-value < 0.05). Both forms of 25(OH)D (total and bioavailable) were positively correlated with Calcium (*p*-value < 0.05) and negatively correlated with iPTH (*p*-value < 0.01).

There were no significant correlations, however, between total and bioavailable 25(OH)D and muscle mass (appSMMI).

To determine the extent to which the 25(OH)D indices are responsible for the variations in the bone density (BUA dB/MHz) and muscle mass (appSMMI kg/m^2^) values, stepwise regression analyses were conducted (Table 5). In this analysis, the variables entered were: age, years since menopause, BMI, BFP, total 25(OH)D, bioavailable 25(OH)D, and free 25(OH)D. For bone density, only age and bioavailable 25(OH)D were significant and explained 7.2% of the variance. For muscle mass, only BMI, BFP and bioavailable 25(OH)D were significant and explained 64.0% of the variance.

**Figure 2:**
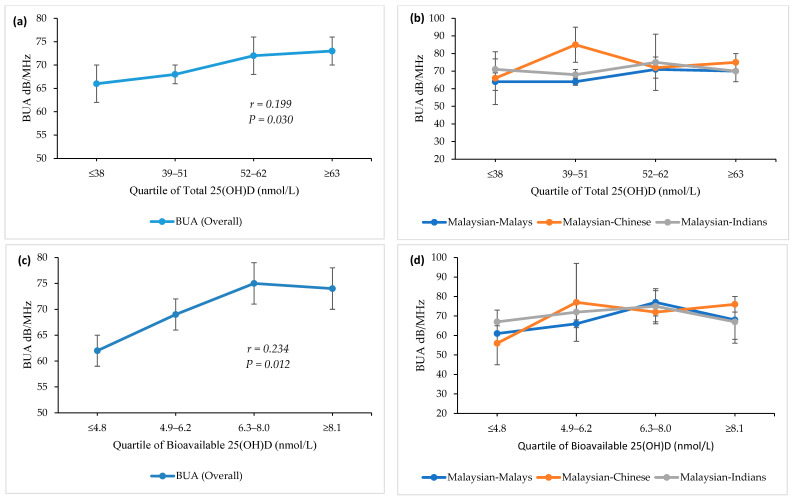
The BUA (broadband ultrasonic attenuation) of participants according to quartiles of Total 25-hydroxyvitamin D: (**a**) The results for all participants; (**b**) the results according to ethnicity: Malaysian-Malays, Malaysian-Chinese and Malaysian-Indians, and Bioavailable 25-hydroxyvitamin D; (**c**) the results for all participants; (**d**) the results according to ethnicity: Malaysian-Malays, Malaysian-Chinese and Malaysian-Indians. Error bar: standard ERROR of mean.

Figure 2 describes the bone density (BUA dB/MHz) of participants at each quartile (from lowest to highest) of total and bioavailable 25(OH)D.

Overall results for participants showed a significant positive correlation between both forms of 25(OH)D and BUA dB/MHz. However, when looking at the differences between ethnicities (Figure 2b,d), results shows that the Malaysian-Chinese reached peak bone density (BUA dB/MHz) at a lower quartile (2nd) compared to the Malaysian-Malays and Malaysian-Indians, which peaked at a higher quartile (3rd).

## 3. Discussion

According to the Institute of Medicine (IOM), the threshold of <30 nmol/L of total 25(OH)D is defined as ‘deficient’ [27]. This threshold was determined by markedly increased parathyroid hormone level or decreased calcium absorption in the body [27,29,30]. However, due to conflicting findings from experimental and epidemiological studies, the optimal threshold for the vitamin D level has not been conclusively defined [31,32]. The IOM committee suggests that the level of serum 25(OH)D that is needed for good bone health for most individuals was 50 nmol/L. Further, the committee suggests that some but not all people with serum 25(OH)D levels between 30 nmol/L and 50 nmol/L are potentially at risk for vitamin D insufficiency [27]. For standardization purposes, the current study had chosen the threshold proposed by IOM (2011) to categorize the vitamin D levels [27].

In the current study, a cohort of Malaysian Malay, Chinese, and Indian postmenopausal women (141 participants), residing in Klang Valley and Semenyih, Malaysia, volunteered to participate in the study (Table 1). However, only 120 participants consented for their blood to be taken and measured for total 25(OH)D, VDBP, intact parathyroid hormone (iPTH), serum calcium and serum albumin. Free and bioavailable 25(OH)D were calculated using the modified Vermuelen method for free testosterone estimation [21]. The Vermeulen method was used because this method gives separate measurements of free and bioavailable 25(OH)D, rather than the Bikle method which only gives the free 25(OH)D [21,33]. Nevertheless, studies have shown that the results of these two formulas are significantly correlated [33]. In this community-dwelling population of postmenopausal Malaysian women, vitamin D deficiency was rare. In fact, few participants had intact parathyroid hormone (iPTH) levels outside the normal range (Table 1, median (IQR) 5.2(3.7) pmol/L). Parathyroid Hormone (PTH) is responsible for maintaining normal levels of calcium and phosphate in the body. The hormone is regulated through the levels of serum vitamin D and calcium [34]. When the level of vitamin D level is high, the level of PTH is low (negative correlation), which was indeed found in the current study and presented in Table 4 [35,36]. In addition to vitamin D deficiency, an elevated PTH concentration is concerning as it is highly associated with cardio-metabolic diseases [37]. Therefore, it is important to monitor both levels of serum 25(OH)D and PTH in general health screening. The median for total 25(OH)D in the current study participants was 50 nmol/L (Table 1). On the basis of the IOM 2011 guidelines, only 7.5% of our participants would be classified as vitamin D deficient (<30 nmol/L, Table 3), while the majority of participants in this cohort were in the vitamin D replete category (51.7%, >50 nmol/L, Table 3). 

According to a 2011 report by the Malaysian Ministry of Science Technology and Innovation (MOSTI), Malaysians normally received at least 6 h of sunshine daily [36]. This high amount of sun exposure may be the reason for their above-average vitamin D levels. Lips also reported a low prevalence of vitamin D deficiency in Southeast Asia, particularly in Malaysia and Singapore [38]. Nevertheless, a number of previous Malaysian studies had shown contrasting findings [36,39]. For example, Shafinaz and Moy [36] reported a high prevalence of vitamin D deficiency among the Malaysian general population (<30 nmol/L), while Moy and Bulgiba [39] reported a high prevalence of vitamin D insufficiency among Malaysian women (<50 nmol/L). The reasons for the discrepancies may be due to differences in age groups, gender, and method of 25(OH)D assessment. The current study, for example, had used the chemiluminescence immunoassay (CLIA) method to assess the total 25(OH)D, which had been proven to be highly sensitive and consistently used for clinical diagnosis [40]. On the other hand, some immunoassays, such as the ones that come in ready-to-use kit versions, may underestimate 25(OH)D metabolites due to the differences in the affinity between the antibodies or D-binding protein employed. Nevertheless, the ethnic disparities in 25(OH)D levels in the current study were consistent with findings of previous Malaysian studies [36,41,42]. The current study found that women of Indian and Malay ethnicity had significantly lower vitamin D level (<50 nmol/L) compared to the Chinese (>50 nmol/L, *p*-value <0.05, Figure 1). Comparatively, Rahman et al. [42] also reported that the level of total 25(OH)D was significantly lower in the postmenopausal Malaysian-Malay women (44.4 [10.6] nmol/L) compared to the Malaysian-Chinese women (68.8 [15.7] nmol/L). 

Studies have consistently found race/ethnicity to be one of the strongest indicators of vitamin D status [36,41,42]. Theoretically, one of the reasons was due to melanin content in the skin. Malaysian-Indians and Malaysian-Malays normally have higher melanin content in the skin (Fitzpatrick skin type VI and types V and VI, respectively) compared to the Malaysian-Chinese (types III and IV) [43]. Higher melanin content inhibits vitamin D synthesis [42]. Studies have found that longer sun exposure is needed by people with darker skin color or higher melanin content in order to produce the same amount of vitamin D levels compared to those with lighter skin color [44]. Although Malaysia is a country with plenty of sunlight all year round, a number of findings showed that the women, particularly of Malay ethnicity, tend to have low vitamin D levels [36,38,39,42]. The Malays constituted about 60% of the country’s population and they are Muslims by religion. Previous studies have shown that Malaysian-Malays tend to stay indoors and avoid the extreme heat of mid-day sun [42]. Although humans only need to be exposed to sunlight for about 15 min during mid-day to obtain adequate vitamin D levels, the clothing habits of Malaysian-Malay women (i.e, headscarf, long sleeves, long skirts) which only exposed the UV radiation to face and hands, as well as darker pigmentation, reduced the synthesis of vitamin D in the skin [38,42]. However, self-identification of ethnicity by participants in the current study posed a limitation, reducing the ability of the current study to make valid conclusion. Participants identified their ethnicity through a simple question in the questionnaire. Further, the skin pigmentation was not directly measured using appropriate spectrometric devices, reducing the ability of the current study to make causal inferences. However, although the ethnicity of the current study’s participants was self-identified, the results of the current study (Figure 1) echoed prior Malaysian studies’ in relation to ethnic disparities in vitamin D levels [36,41,42]. In the case of free and bioavailable 25(OH)D, the levels of these two forms of 25(OH)D reflects the level of total 25(OH)D (Figure 1 and Table 4). Collectively, the median for bioavailable 25(OH)D for this study cohort was 2.5 ng/mL (Table 1), comparable to previous findings in healthy women in Seoul, Korea (2.6 ng/mL) [10] and Shanghai, China (2.9 ng/mL) [45]. 

Current and other studies have shown that obesity was negatively associated with serum total 25(OH)D level [45]. However, it was unclear which indicator of obesity, i.e., BMI, waist circumference, (WC) or percentage of body fat (BFP) should be taken into consideration while assessing vitamin D status in the general population. The current study found no differences in BMI and WC between the vitamin D deficient, insufficient, and the replete group (Table 3). Interestingly, findings from the current study suggest that there may be a certain degree of body fat percentage (BFP) that may trigger the negative association with vitamin D. For example, the current study found that the BFP of people in the Replete group (38.8%) was significantly lower (*p*-value < 0.05) than the Deficient and Insufficient groups (43.6% and 43.1%, respectively, Table 3), although it was still above the threshold for osteopenic-related obesity level (BFP ≥32%) [24]. 

When compared with vitamin D deficient group, people in the vitamin D replete group had significantly lower iPTH level, and significantly higher bone density (BUA) and faster walking speed (Table 3, *p*-value < 0.05). The vitamin D replete group also had significantly higher serum calcium and bioavailable 25(OH)D concentration than vitamin D deficient group (Table 3). Between the three groups of vitamin D deficient, insufficient, and replete, there were no significant differences in the HGS, muscle mass, lower extremity strength (sit-to-stand test), balance, and VDBP (Table 3). Nevertheless, vitamin D has been described to have a U-shaped relationship with the physical function where plasma 25(OH)D levels greater than 120 nmol/L was found to be associated with a poorer physical performance [46]. Therefore, non-significant differences between the groups may not indicate a lack of correlations. Perhaps a dose-dependent study could confirm the U-shaped relationship.

### 3.1. Vitamin D and Bone Density

Studies have consistently found a significant and positive relationship between vitamin D and bone health [47]. Vitamin D increases the absorption of calcium in the gut, regulates the mineralization of bone tissue and may play an important role in muscle function [48]. Recent evidence suggests that the unbound or the bioavailable fraction of 25(OH)D may be a better indicator of vitamin D status compared to total 25(OH)D as it has been shown to correlate better with bone density [45]. The free hormone hypothesis posits that the free and/or bioavailable fractions of 25(OH)D may correlate more strongly with its biological action than the total 25(OH)D due to their higher biological ‘availability’ [49]. Currently, there are limited assessments of bioavailable 25(OH)D in the Asian population. So far, there was only one other study which had examined the free hormone hypothesis related to 25(OH)D in a cohort of Malaysian women [33]. However, the study was conducted on women with rheumatoid arthritis (mean age 53.7 years) and the results may not be applicable to the general, community-dwelling population.

In the current cross-sectional study, Pearson’s correlation coefficient analysis revealed that both the bioavailable 25(OH)D and total 25(OH)D levels were positively correlated with the BUA (Table 4 and Figure 2, *p*-value < 0.05). Interestingly, when divided by ethnicity, the Malaysian-Chinese achieved peak (highest) BUA at a lower concentration of total 25(OH)D (Figure 2) and bioavailable 25(OH)D (Figure 2) compared to Malaysian-Malays and Malaysian-Indians (second quartile vs. the third quartile). The reason for this may be due to existing calcium concentrations. A prior study has reported that calcium-sufficient people need less vitamin D [47]. Heaney [47] found that African-Americans had better renal conservation of calcium (more efficient calcium economy) compared to Caucasians, which explains why, despite their low vitamin D level, African-Americans have a lower risk of osteoporotic fractures than do Caucasians [50]. Indeed, in the current study, the Malaysian-Chinese had significantly higher calcium concentration compared to Malaysian-Malays and Malaysian-Indians (Figure 1), thus do not need as much vitamin D to aid calcium absorption in the gut for bone remodeling (i.e., higher BUA at a lower levels of vitamin D). 

In the Stepwise regression analyses, the current study identified age and bioavailable 25(OH)D, (but not total 25(OH)D) as significant predictors for BUA and these two variables explained 7.2% of the variance (Table 5). Age was found to be negatively correlated (β = −0.191), while bioavailable 25(OH)D (β = 0.267) was positively correlated with bone density. Interestingly, even without any adjustment of variables, the Pearson’s correlation coefficient of bioavailable 25(OH)D to the BUA was still marginally stronger compared to total 25(OH)D [*r* = 0.234, *p*-value = 0.012 and *r* = 0.199, *p*-value = 0.030, respectively, Figure 2a,c]. These findings support the findings by Li et al. [45] which found that bioavailable 25(OH)D levels (but not total 25(OH)D levels) were an independent determinant of the bone mineral density (BMD) values after adjusted for age, body mass index, and bone turnover biomarkers (OST and β-CTX) in postmenopausal women. We also found that there was a slightly stronger correlation between the iPTH concentrations and bioavailable 25(OH)D than with the total 25(OH)D level (Table 4). However, in the current study, the small effect size (Pearson’s r) and the small percent of variance are insufficient to conclude the superior status of bioavailable 25(OH)D. More research is needed with a larger sample size to validate this finding.

To date, several epidemiological studies have determined the correlation between levels of different forms of 25(OH)D and bone density in healthy populations, but inconclusive results were found. A study by Johnsen et al. [49] on the correlations between different forms of 25(OH)D and BMD found that bioavailable or free 25(OH)D was the superior indicator for vitamin D assessment compared to total 25(OH)D [49]. Conversely, a study with 304 adults aged between 21 and 81 years found no significant association between any form of 25(OH)D and BMD [51]. In the current study, our findings supported the former (Table 5). In addition to BUA, we also found a significant and positive correlation of bioavailable 25(OH)D with other QUS indices (results reported elsewhere). When age, years since menopause, obesity index (BMI and BFP), and 25(OH)D index were tested as predictors, the strongest correlations to BUA were found for age and bioavailable 25(OH)D, suggesting the superior role of the metabolite compared to total 25(OH)D (Table 5). This is somewhat supported the findings by Lim et al. [52], who found that increased age was positively associated with osteoporosis among healthy Malaysian women (≥45 years). In addition, Chan et al. [53] also found that the predictors of suboptimal bone health and osteoporosis among 786 Malaysians aged > 40 years were increased age and higher fat mass. Interestingly, we found no evidence for the obesity paradox which hypothesized that obesity is correlated with higher bone density (Table 4). No significant correlations were found between indicators of obesity (BMI and BFP) and BUA (Table 4 and Table 5), suggesting that women with higher adiposity may not have higher bone density. This is consistent with previous studies reporting no correlations between obesity and high BMD [53]. However, it is fair to note that for obese populations, there is a U-shape relationship between BMI and BMD and that the protective effects of weight on bone are reduced along with the increment of BMI [54]. Palermo et al. [54] found that the protective effects of weight on bone only went up to a certain level, and decreased along with the increment of BMI. So far, many studies exploring the relationships between obesity and bone density in human subjects had been observational in nature. Further studies with identifiable confounding factors are needed in order to determine the impact of obesity on bone density. 

### 3.2. Vitamin D and Muscle Mass

In muscle, the active form of vitamin D was theorized to stimulate muscle cell proliferation and growth by activating vitamin D receptors (VDR) that mediate both gene transcription and rapid non-transcriptional signal transduction. This, in turn, will regulate protein synthesis and calcium handling involved in muscle cell development [55]. Vitamin D has been found to play a significant role in muscle function in a number of studies [56,57]. For example, a Korean study involving adults aged 40 years and older showed that men with vitamin D deficiency (defined as serum 25(OH)D <  20 ng/mL) had lower appendicular skeletal muscle mass (appSMMI) than those with a higher level of vitamin D. No correlation, however, was found in women [57]. Other studies have found similar findings. For example, Visser, Deeg & Lips [58] found that after adjustment for physical activity level, the season of data collection, serum creatinine concentration, chronic disease, smoking, and BMI, older adults with low baseline 25(OH)D levels (<25 nmol/L) were 2.14 times (0.73–6.33, based on appendicular skeletal muscle mass) more likely to experience sarcopenia compared to those with high 25(OH)D levels (>50 nmol/L). Interestingly, in the current study, stepwise regression analysis revealed a significant and positive correlation between bioavailable 25(OH)D (but not total 25(OH)D) and appSMMI (Table 5, *p*-value < 0.10). Similar to bone density (BUA), bioavailable 25(OH)D was found to be superior to total 25(OH)D in its correlation to muscle mass (appSMMI, Table 5). However, more than bioavailable 25(OH)D, a big proportion of the appSMMI variance was explained by the obesity indices, which includes BMI and BFP (*p*-value < 0.05, Table 5). Indeed, the current study found a significant and positive correlation between obesity (BFP) and muscle mass (appSMMI, Table 4). It is entirely possible that vitamin D may play a role in the parallel increase of muscle mass along with fat. Whilst vitamin D is primarily stored in adipose tissue, there is evidence of vitamin D uptake in skeletal muscle [59]. Vitamin D uptake in skeletal muscle may help the muscle grow along with body fat as a part of body’s adaptation to compensate for the increase in body mass (i.e., Davis’s law, which is a corollary to Wolff’s Law, describes how soft tissue increase according to the manner in which they are mechanically stressed) [60]. 

In addition to a deficit in muscle mass, hypovitaminosis D has also been found to be significantly associated with a decrease in lower limb strength in older men and women [9]. A cross-sectional study conducted by Ahern et al. [61] reported slower walking speed in 252 severely obese and vitamin D deficient adults [61]. In the current study, we found that different forms of 25(OH)D are correlated with different components of functional performance (Table 4). For example, total 25(OH)D was found to be significantly and positively correlated with walking speed (GS), whereas bioavailable 25(OH)D was found to be correlated with lower extremity strength (STS, Table 4, *p*-value < 0.05). These observed associations suggest that regardless of fractions of vitamin D, older women with low vitamin D level may be at a higher risk for future falls and fall-related injury due to weaker lower body strength. Other studies have also reported significant associations between low vitamin D status and subsequent falls risk, a decline in physical performance and development of sarcopenia in the elderly [58,62,63]. 

## 4. Materials and Methods 

### 4.1. Selection and Recruitment of Participants

One hundred and forty-one (*n* = 141) postmenopausal women comprised of Malay, Chinese and Indian ethnicity (aged 45–88 years) residing in Klang Valley and Semenyih, Malaysia volunteered to participate in the study. Of which, one hundred and twenty (*n* = 120) women consented to their blood being collected and analyzed for intact parathyroid hormone (iPTH), albumin, calcium, vitamin D binding protein (VDBP), and total 25(OH)D. One sample for iPTH was excluded from analysis due to being an outlier and 4 samples for VDBP were excluded due to the high percentage of coefficient variations (>15% CV). Free and bioavailable 25(OH)D were calculated using a modified Vermuelen method for free testosterone estimation [21,64]. Postmenopausal was defined as having no menstrual period, bleeding, or spotting during the 12 consecutive months prior to enrollment, and was assessed using a questionnaire. Before enrollment, details about the study covering the objectives, procedures, benefits, risks, and possible discomforts from the study were briefed to interested participants. Apparently healthy and interested participants were screened for eligibility with the following inclusion criteria: (i) A woman, (ii) Citizen of Malaysia (of Malay, Indian or Chinese ethnicity), (iii) Postmenopausal (no menstrual period, bleeding, or spotting 12 consecutive months prior to enrolment). Exclusion criteria included: (i) Inability to stand for height, weight and gait speed assessments, (ii) Presence of artificial limbs and/or metal implants, (iii) Severe cardiac, pulmonary, or musculoskeletal disorders, (iv) Severe cognitive impairment or any disability that makes communication impossible, and (v) Presence of terminal illness. Eligible participants were explained further on the purpose and procedure of the study and were asked to give written consent. This study was conducted according to the guidelines laid down in the Declaration of Helsinki and all procedures involving research study participants were approved by the Science and Engineering Research Ethics Committee of the University of Nottingham Malaysia [SEREC- NZA051016].

### 4.2. Demographic Status

Demographic information was collected using a structured and validated questionnaire with items including age, sex, level of education, history of diseases/co-morbidities, and self-rated level of physical activity. Questions on menstrual status were taken from the Menopause Health Questionnaire, The North American Menopause Society (e.g., ‘how would you describe your current menstrual status?’ with options to choose pre-menopause, peri-menopause and post-menopause, each provided with definitions).

### 4.3. Anthropometric and Obesity Indices Measurements

#### 4.3.1. Height 

Height was measured to the nearest 0.1 cm using a portable stadiometer (SECA 217, Vogel & Halke GmbH & Co., Hamburg, Germany). Participants were asked to stand with their shoulders, buttocks and heels resting against the stadiometer, toe tips forming a 45° angle, heels touching each other, head held straight and neck in a natural position.

#### 4.3.2. Body Fat Percentage and Body Mass Index

Body fat percentage and body mass index was assessed using a segmental bio-electrical impedance analyzer (InBody 230 Body Composition Analyzer, Biospace Co. Ltd., Seoul, Korea). While on this machine, the weight of the participant was automatically generated.

#### 4.3.3. Waist Circumference

A measuring tape (SECA 203, GmbH & Co. Kg., Hamburg, Germany) was used to measure waist circumference. Waist circumference (cm) was measured at the mid-point between the last rib and the anterior superior iliac spine with subjects standing upright [23].

### 4.4. Bone Density Index Measurement

#### Quantitative Ultrasound (QUS) Bone Assessments 

Bone density was assessed using calcaneal ultrasound bone densitometer (SAHARA^®^ Clinical Bone Sonometer, Hologic Inc, Waltham, MA, USA). Prior studies using quantitative ultrasound (QUS) found that high-frequency sound waves were attenuated easier by bone compared to low-frequency sound waves. Ultrasonic sound waves in the frequency range of 0.2 to 0.6 MHz were found to be linearly correlated with the level of attenuation. The slope of the linear regression of these two parameters (attenuation versus sound waves in the frequency range) was defined as broadband ultrasound attenuation (BUA), and is measured in dB/MHz. On the SAHARA^®^ system, the BUA and Speed of Sound (SOS) are measured simultaneously. In order to determine the sound attenuation of the heel alone, without any bias arising from the transducers and/or transducer pads, a comparison measurement was made through a reference medium (SAHARA^®^ QC Phantom supplied with the SAHARA^®^ unit when the unit was calibrated at the factory). While SAHARA^®^ densitometers do not directly measure bone mineral density (BMD), the BUA and SOS results are correlated (*r* = 0.82–0.85) with heel BMD results obtained by the standard Dual Energy X-ray Absorptiometry (DXA) technique [65]. 

### 4.5. Muscle Mass Indices Measurements

#### Appendicular Skeletal Muscle Mass Index (appSMMI)

Muscle mass indices [skeletal muscle mass (SMM), fat-free mass (FFM) and appendicular skeletal muscle mass (appSMM)] were assessed using segmental bio-electrical impedance analyzer (BIA, InBody 230 Body Composition Analyzer, Biospace Co. Ltd., Seoul, Korea). Appendicular skeletal muscle mass was calculated by adding the weight of the muscle masses of the four limbs. Appendicular Skeletal Muscle Mass Index (appSMMI) was defined as the sum of the muscle masses of the four limbs, adjusted for height in squared meters (kg)/height^2^. AppSMMI was first suggested by Baumgartner et al. [66] in the New Mexico Elder Health Survey. This index provided significant associations with physical disability or frailty.

### 4.6. Muscle Strength

Handgrip strength was assessed as a proxy for muscle strength and was measured in each hand using a hand dynamometer (JAMAR Hydraulic Hand Dynamometer^®^ Model PC-5030 J1, Fred Sammons, Inc., Burr Ridge, IL, USA). Handgrip strength was measured twice for each hand, and the higher of the two values was recorded. Then, the higher value of the two hands was used in the analysis. Standardized positioning recommended by The American Society of Hand Therapists (ASHT) was used: subject seated, shoulders adducted and neutrally rotated, elbow flexed at 90°, forearm in neutral and wrist between 0 and 30° of dorsiflexion [67].

### 4.7. Functional Performance

#### 4.7.1. Short Physical Performance Battery (SPPB) Test

Functional performance was assessed using modified components of the short physical performance battery (SPPB) test. Based on the recommendation by Ilich, Kelly and Inglis [24], the following tests were conducted under the SPPB: one-leg stance (to test balance), gait speed (to test endurance), and sit-to-stand chair test (to assess lower extremity strength). The SPPB has an internal consistency of 0.76 and has predictive validity for the risk of mortality, nursing home admission, and disability [68]. 

##### 4.7.1.1. One-Leg Stance

For the one-leg stance, measurements for both the right and left legs were assessed. The test requires participants to stand on one leg while lifting the other limb, for a maximum of 30 s. The test stops when the participant touches any surface or lowers the other limb to the ground or, ultimately, at the end of 30 s [24]. 

##### 4.7.1.2. Gait Speed

Gait speed was measured by timing a 6-m normal walk. The 6-m course was marked by two cones or pieces of tape measured using a roll-up, self-retracting construction measuring tape. The test requires the participant to walk at a normal pace starting at one end of the course and all the way past the other. The timing starts when the tester/instructor command “begin” and stops when one of the participant’s feet is all the way across the 6-m marker. If the participants normally use a cane or any other walking devices, they were allowed to use them while performing the test. 

##### 4.7.1.3. Sit-to-Stand Chair Test

At the beginning of this test, the participant was seated in an armless chair, with arms crossed over the chest, back straight, and feet flat on the floor. At the maximum of 30 s, the test requires the participant to rise from the chair and sit down again as many times as possible. The number of consecutive chair sit-to-stand tests completed was recorded, with the last time the participant sat down in the chair being the final count. 

### 4.8. Laboratory Measurements

Blood samples were collected by a trained phlebotomist, processed and stored at −80 °C until measurement. Blood serum was collected using yellow-topped plasma tubes (SST) and plasma was collected using lavender-topped plasma tubes (EDTA as additive). All blood samples were centrifuged at 1200× *g* for 15 min, aliquoted, and stored at −80 °C until measurement. Serum VDBP was measured using ELISA technique [Quantikine ELISA kit (R&D Systems, Minneapolis, MN, USA, Product code: GZ-DVDBP0B)] that employs quantitative sandwich enzyme immunoassay using a monoclonal antibody. The intra-assay CV was 5.4%. The % recovery was 100.13% (MyAssays.com). Serum total 25(OH)D concentrations were measured using chemiluminescent microparticle immunoassay CMIA on Siemens^®^ platforms (Siemens AG, Munich, Germany). Serum albumin levels were measured by using BCG Dye bonding on ADVIA^®^ 2400 Clinical Chemistry System (Siemens AG, Munich, Germany), plasma intact parathyroid hormone levels was measured using 2-site sandwich microparticle immunoassay on Siemens^®^ ADVIA^®^ Centaur XP immunoassay system (Siemens AG, Munich, Germany), and serum calcium level was measured using Arsenazo III Method on ADVIA^®^ 2400 Clinical Chemistry System (Siemens AG, Munich, Germany). Free and bioavailable 25(OH)D were calculated from total 25(OH)D, VDBP, and serum albumin concentrations using Vermuelen method for free testosterone estimation [16,21,64,69,70] (Supplementary Material 1):

Bioavailable 25(OH)D = free 25(OH)D + albumin bound 25(OH)D(1)

(2) $$\text{Free}\;25(\text{OH})\text{D}=[-\text{b}+\sqrt{{\text{b}}^{2}-4\text{ac}}]÷2\text{a}$$

Bioavailable 25(OH)D = [Free 25(OH)D] + [DAlb] = (K_alb_ × [Alb] +1) × Free 25(OH)D (3)

a = K_VDBP_ × K_alb_ × [Alb] + K_VDBP_ (4)

b = (K_VDBP_ × [VDBP])–(K_VDBP_ × [Total 25(OH)D]) + (K_alb_ × [Alb]) + 1 (5)

c = − [Total 25(OH)D](6)

K_alb_ = affinity constant between 25(OH)D and albumin = 6 × 10^5^ M^−1^ (7)

K_VDBP_ = affinity constant between 25(OH)D and VDBP = 7 × 10^8^ M^−1^ (8)

[Alb] = concentration of albumin(9)

[Total 25(OH)D] = concentration of total 25(OH)D(10)

[VDBP] = concentration of VDBP(11)

[DAlb] = Albumin-bound 25(OH)D = Bioavailable 25(OH)D−Free 25(OH)D(12)

Calculations of all forms of 25(OH)D were done in moles per liter (mol/L) using the Vermeulen method as it provides separately for free and bioavailable 25(OH)D. Subsequently, bioavailable 25(OH)D was converted to nanomoles per litre (nmol/L) while free 25(OH)D was expressed as nmol/L and picomoles per litre (pmol/L) (please refer to the Supplementary Material 1 for the conversion table).

### 4.9. Statistical Analysis

Statistical analyses were performed using IBM SPSS (Statistical Package for Social Sciences) version 24 for Windows (SPSS, Inc., Chicago, IL, USA). Variables were checked for normality (Shapiro–Wilk test) and presented as mean ± standard deviation or median (interquartile range). Where possible, analyses were stratified by ethnicity. Frequency and percentages were reported for categorical variables. Outliers were detected using ‘outlier labeling rule’, which is based on multiplying the Interquartile Range (IQR) by a factor of 2.2 to get the higher or lower range of the data [71]. A comparison of the distributions of various parameters between groups was performed using a standard ANOVA (analysis of variance) or ANOVA’s Welch Test (dependent on the smallest number of sample size). When significant differences were found with ANOVA, the post-hoc Tukey’s HSD (honestly significant difference) or Games-Howell test was applied to correct for use of multiple comparisons. Pearson’s correlation analysis was used to assess the correlation of each of the characteristics with the target outcomes. Stepwise regression analysis was used to determined significant predictors for bone density and muscle mass. Two-tailed *p*-value ≤ 0.05 was recognized as statistically significant, unless stated otherwise. 

## 5. Conclusions

Although in the current study, bioavailable 25(OH)D was found to be superior to total 25(OH)D in its correlation to bone density (BUA) and muscle mass (appSMMI), it is still premature to draw any conclusion on the relationship due to the small effect size (R^2^) and Pearson’s r value. More research is needed with a larger sample size in order to validate these findings. Nevertheless, the significant correlation found between bioavailable 25(OH)D levels and bone density shows that it may be helpful in clinical practices when assessing bone health in postmenopausal women. Experts generally agree that a minimum level of 50  nmol/L of 25(OH)D must be reached in the general elderly population and that 75  nmol/L should be the target in fragile people who are at elevated risk for falls and fractures [72,73]. Vitamin D supplementation is generally safe and inexpensive. Therefore, it is highly recommended in patients at risk for falls, such as the elderly, institutionalized patients, frail patients, and patients with chronic diseases. These individuals tend to have low levels of vitamin D and muscle disorders. Therefore, supplementation is justified regardless of any assumed effect on the prevention of falls. Further, the current study shows evidence on the negative implication of obesity on total vitamin D levels, and that the levels of bioavailable 25(OH)D is dependent on the levels of VDBP. This study is worth further exploration. The findings could revolutionize the testing for vitamin D status in the general population, particularly in vulnerable groups such as the obese.

### 5.1. Limitations

This study has some limitations. First, the cross-sectional design of the study prevents us from making any causal inferences. Second, skin pigmentation was not directly measured, limiting our ability to validate the differences in 25(OH)D levels between ethnicities. For future studies, it is better to measure the variability of skin pigmentation using a measurement device such as spectrophotometer (Konica Minolta CM700d) or telespectroradiometer (PhotoResearch PR650). Third, ethnic classification was based on self-identification (asked on the questionnaire as ‘What is best described as your ethnicity?’), and therefore is considered as non-scientifically accurate ethnic classification. Nevertheless, self-identification of ethnicity has always been used even in Malaysian national census and Malaysians are generally used to providing information on their ethnicity [74]. Further, genotype-specific affinity constants were not used in the calculation of free and bioavailable 25(OH)D due to unavailable data on VDBP polymorphisms in the Malaysian population. This may be a confounding factor. Nevertheless, Li et al. [45] suggest that the VDBP variants account for only a small proportion of the bioavailable 25(OH)D variation as they found no associations between the VDBP level and the presence of VDBP variants rs4588 and rs7041 with bone density. However, there is merit in studying the Group-specific Component (GC) phenotype due to evidence showing a high degree of polymorphism in VDBP [75]. Genetically, VDBP is highly polymorphic with three frequent alleles (DBP/GC 1f, 1s, and 2) and over 120 variants [75]. Currently, the health consequences of this polymorphism are not yet understood. Therefore, there is a need to standardize the assays for serum VDBP so that the role of VDBP in the manifestation of diseases can be determined. Finally, the current study had used ultrasound and bio-electrical impedance analyzer to measure bone density and muscle mass. For future studies, it is better to use the gold standard spectral imaging such as Dual-energy X-ray absorptiometry (DEXA) as the measurement tool for bone density and body composition in order to increase the validity of the study. 

### 5.2. Strengths 

Notwithstanding the above limitations, the current study had several strengths. One of the strengths of this study lies in the representativeness of the study sample which includes the 3 major ethnicities in Malaysia (e.g., Malaysian-Malays, Malaysian-Chinese and Malaysian-Indians), which described the differences of vitamin D levels of multi-races with similar environments, crediting the external validity of the results. Further, the older participants were non-institutionalized, allowing direct extrapolation to older persons of the population at large. In addition, we determined the better biomarker between total and bioavailable 25(OH)D levels in relation to bone and muscle indices, which could lead to the elucidation of the underlying mechanisms for the effects of 25(OH)D on osteoporosis and sarcopenia. 

## Figures and Tables

**Table 1:** Characteristics (Malay, *n* = 51; Chinese, *n* = 42; Indian, *n* = 48) and blood parameters (Malay, *n* = 49; Chinese, *n* = 35; Indian, *n* = 36) of participants.

Variables		Reference	*N*	Mean ± SD/Median (IQR)	Minimum–Maximum
Age (years)			141	59.0(10.0)	45.0–88.0
Age at menopause (years)			121	50.0(6.0)	36.0–59.0
Years since menopause			121	8.0(11.0)	1.0–35.0
Height (cm)			141	153.1 ± 6.2	137.5–169.0
Weight (kg)			141	63.4 ± 12.6	31.9–100.9
BMI (kg/m^2^) ^δ^	All		141	27.1 ± 5.3	15.4–43.0
	Normal	18.5–22.99	29	20.8 ± 1.3	18.5–22.8
	Overweight	23.0–27.49	40	25.0(2.65)	23.1–27.4
	Obese Type 1 and 2	≥27.5	68	30.25(4.1)	27.5–43.0
Waist circ. (cm) ^µ^	All		139	84.2 ± 12.6	55.1–121.0
	Overweight/Obese	≥80 cm	87	89.7(11.2)	80.0–121.0
Body fat (%)	All		141	41.8(10.45)	20.7–54.0
	Obese ^γ^	≥32%	121	43.1(8.8)	32.3–54.0
AppSMMI (kg/m^2^)	All		140	6.1(1.1)	4.0–10.7
	Sarcopenic ^β^	≤5.7 kg/m^2^	44	5.3(0.4)	4.01–5.69
BUA (dB/MHz)	All		139	70.0 ± 16.8	35.9–122.2
	Osteopenic ^α^	<54 dB/MHz	25	47.5(9.75)	35.9–53.8
Total 25(OH)D (nmol/L) ^ƒ^		>50 nmol/L	120	51.0(23.85)	23.0–117.6
Serum Calcium (mmol/L) ^ƚ^		2.10–2.55 mmol/L	120	2.4 ± 0.1	2.15–2.65
Plasma iPTH (pmol/L) ^ƚ^		1.5–7.6 pmol/L	119	5.2(3.7)	1.60–12.30
Serum albumin (g/L) ^ƚ^		35–50 g/L	120	45.0(5.0)	38.0–50.0
Bioavailable 25(OH)D (nmol/L)			116	6.2(3.2)	2.83–17.0
Bioavailable 25(OH)D (ng/mL) ^ƚ^		1.92–8.82 ng/mL	116	2.5 (1.3)	1.13–6.80
Free 25(OH)D (pmol/L)			116	15.7(7.4)	6.87–43.2
VDBP (ug/mL) ^ƚ^		104–477 ug/mL	116	224.7 ± 44.8	123.5–327.7

N.B: appSMMI = appendicular skeletal muscle mass index, BMI = body mass index, four participants (*n* = 4) were underweight BMI < 18.5 kg/m^2^, BUA = broadband ultrasonic attenuation, SD = standard deviation, iPTH = intact parathyroid hormone, IQR = interquartile range, VDBP = vitamin D binding protein, 25(OH)D = 25-hydroxyvitamin D, ^δ^ = WHO, 2004 [22], ^µ^ = WHO, 2008 [23], ^γ^ = Ilich et al., 2016 [24], ^β^ = AWGS [25], ^α^ = Johansen, Evans & Stone, 1999 [26], ^ƒ^ = based on cut-points by IOM (Institute of Medicine) Dietary Reference Intakes for Calcium and Vitamin D. Washington, DC: The National Academies Press; 2011 [27], ^ƚ^ Pan Laboratories, Irvine, USA reference range for adults, http://panlaboratories.com/bioavailable-vitamin-d-25-hydroxy/which were based on previous studies [19,21,28].

**Table 2:** The demographic data of study participants.

Variables	N Total	Breakdown of variables	*n*	Percentage (%)
Education Levels	140	No Formal Education	10	7.1
		Primary School	22	15.7
		Secondary School	55	39.3
		Certificate/Diploma	29	20.7
		University Degree	19	13.6
		Postgraduate Degree	5	3.6
Cigarette Smoking Status	139	Non-smoker	137	98.6
		Current smoker	2	1.4
Alcohol Drinking	138	Non-drinker	136	97.8
		Current drinker	2	2.2
Self-rated PA Status	137	Inactive	69	50.4
		Active (at least 10 mins per day)	68	49.6
Disease(s)/disorder(s)	139	None	60	43.2
		Hypertension	53	
		Diabetes Type 2	31	
		Heart problems	11	
		Osteoarthritis	12	
		Rheumatoid Arthritis	7	
		Osteoporosis	8	
		Have had stroke	4	
		Depression/anxiety	6	

N.B: PA = physical activity.

**Table 3:** The differences in characteristics for participants who were Vitamin D (Total 25(OH)D) deficient, insufficient, and replete.

Variables	Deficient (<30 nmol/L)^ƒ^ Mean (SD), *n* = 9	Insufficient (30–50 nmol/L)^ƒ^ Mean (SD), *n* = 49	Replete (>50 nmol/L)^ƒ^ Mean (SD), *n* = 62	*p*-Value *
HGS (kg)	20.7 (5.5)	18.7 (4.7)	20.8 (4.6)	0.091
AppSMMI (kg/m^2^)	6.0 (1.3)	6.1 (0.8)	6.1 (0.7)	0.978
SMMI (kg)	8.7 (1.5)	8.4 (1.0)	8.4 (0.9)	0.822
FFMI (kg)	16.3 (2.3)	15.8 (1.6)	15.7 (1.5)	0.695
BFP (%)	43.6 (9.8) **	43.1 (6.7) **	38.8 (8.1)	**0.020**
BMI (kg/m^2^)	29.9 (7.5)	28.3 (5.5)	26.1 (4.9)	0.073
WC (cm)	89.0 (14.8)	84.6 (12.6)	83.9 (12.4)	0.625
BUA (dB/MHz)	56.5 (16.6) **	68.5 (15.8)	72.2 (17.4)	**0.047**
Sit-to-stand test (times in 30 s)	11.8 (2.6)	11.1 (3.7)	12.4 (3.6)	0.213
Walk speed (m/s)	0.7 (0.2) **	0.9 (0.2) **	1.0 (0.3)	**0.010**
Balance (sec)	17.9 (11.7)	17.3 (11.8)	21.3 (9.8)	0.200
Calcium (mmol/L)	2.3 (0.1) **	2.4 (0.1)	2.4 (0.1)	**0.015**
iPTH (pmol/L)	9.1 (3.1) **	6.7 (2.5) **	4.7 (2.2)	**0.000**
Bioavailable 25(OH)D (nmol/L)	3.6 (0.6) **#	5.3 (1.2) **	8.8 (3.0)	**0.000**
Free 25(OH)D (pmol/L)	9.2 (1.6) **#	13.2 (3.1) **	22.0 (7.0)	**0.000**
VDBP (ug/mL)	206.1 (36.8)	227.1 (48.3)	225.6 (43.0)	0.328

N.B: appSMMI = appendicular skeletal muscle mass index, BFP = body fat percent, BUA = Broadband ultrasonic attenuation, FFMI = fat free mass index, HGS = handgrip strength, iPTH = intact parathyroid hormone, SD = standard deviation, SMMI = skeletal muscle mass index, WC = waist circumference, VDBP = vitamin D binding protein, 25(OH)D = 25-hydroxyvitamin D, ƒ = based on cut-points by IOM (Institute of Medicine) Dietary Reference Intakes for Calcium and Vitamin D. Washington, DC: The National Academies Press; 2011 [27], * analysed using one-way ANOVA (Welch Test) with Games-Howell Post-hoc test, *p*-value ≤ 0.05 (indicated in bold), ** = different from Replete, # = different from Insufficient.

**Table 4:** Pearson’s correlation coefficient of blood biomarkers with fat, bone, muscle, and functional performance.

Parameters	Total 25(OH)D (nmol/L)	Bio 25(OH)D (nmol/L)	VDBP (ug/mL)	Calcium (mmol/L)	iPTH (pmol/L)	BFP (%)	BUA (dB/MHz)	AppSMMI (kg/m^2^)	STS	GS (m/s)
**Total 25(OH)D**	1	0.883 **	−0.045	0.192*	−0.394 **	−0.298 **	0.199 *	0.012	0.129	0.191 *
**Bio 25(OH)D**	0.883 **	1	−0.446**	0.238*	−0.426 **	−0.380 **	0.234 *	−0.007	0.202 *	0.134
**VDBP**	−0.045	−0.446 **	1	0.123	0.071	0.149	−0.080	0.124	0.002	−0.115
**Calcium**	0.192 *	0.238 *	0.123	1	−0.497 **	−0.294 **	0.072	−0.170	0.164	0.045
**iPTH**	−0.394 **	−0.426 **	0.071	−0.497**	1	0.448 **	−0.117	0.241 **	−0.249 **	−0.067
**BFP**	−0.298 **	−0.380 **	0.149	−0.294**	0.448 **	1	0.043	0.359 **	−0.198 *	−0.046
**BUA**	0.199*	0.234 *	−0.080	0.072	−0.117	0.043	1	0.192 *	0.143	0.050
**AppSMMI**	0.012	−0.007	0.124	−0.170	0.241 **	0.359 **	0.192 *	1	0.011	0.248 **
**STS**	0.129	0.202 *	0.002	0.164	−0.249**	−0.198 *	0.143	0.011	1	0.350 **
**GS**	0.191 *	0.134	−0.115	0.045	−0.067	−0.046	0.050	0.248 **	0.350 **	1

N.B: appSMMI = appendicular skeletal muscle mass index, BUA = broadband ultrasonic attenuation, BFP = body fat percent, GS = gait speed, iPTH = intact parathyroid hormone, STS = sit-to-stand, VDBP = vitamin D binding protein, 25(OH)D = 25-hydroxyvitamin D, * *p*-value < 0.05, ** *p*-value < 0.01.

**Table 5:** Stepwise regression analyses of vitamin D indices with bone density (BUA) and appendicular skeletal muscle mass index (appSMMI).

Dependent Variables	Predictors	Significant Predictors	Beta	*t* Value	Significance	R^2^	Adjusted R^2^	F
BUA (dB/MHz)	Age (years)	Age	−0.191	−1.939	0.055 *	0.090	0.072	4.774 **
	Years since menopause	Bio 25(OH)D	0.267	2.700	0.008 ***			
	BMI (kg/m^2^)							
	BFP (%)							
	Total 25(OH)D (nmol/L)							
	Bio 25(OH)D (nmol/L)							
	Free 25(OH)D (pmol/L)							
AppSMMI (kg/m^2^)	Age (years)	BMI	1.326	11.665	0.000 ***	0.650	0.639	58.821 ***
	Years since menopause	BFP	−0.716	−6.112	0.000 ***			
	BMI (kg/m^2^)	Bio 25(OH)D	0.120	1.824	0.071 *			
	BFP (%)							
	Total 25(OH)D (nmol/L)							
	Bio 25(OH)D (nmol/L)							
	Free 25(OH)D (pmol/L)							

N.B: BFP = body fat percent; BMI = body mass index; BUA = broadband ultrasonic attenuation; 25(OH)D = 25-hydroxyvitamin D; appSMMI = appendicular skeletal muscle mass index. * Significance at *p*-value < 0.1 ** Significance at *p*-value < 0.05, *** Significance at *p*-value < 0.01.

## Data Availability

The data presented in this study are openly available in FigShare at https://doi.org/10.6084/m9.figshare.13317857.v1.

## Supplementary Materials

The following are available online at https://www.mdpi.com/2218-1989/11/1/23/s1, Supplementary Material 1: Calculations of Free and Bioavailable 25-hydroxyvitamin D based on Vermeulen’s equations, Table S1: Conversion table for free and bioavailable 25(OH)D calculations.
