# Supplementary material for: Total vs. Bioavailable: Determining a Better 25(OH)D Index in Association with Bone Density and Muscle Mass in Postmenopausal Women
Source: Metabolites. 2020 Dec 31;11(1):23. doi: 10.3390/metabo11010023 (PMC7824471; doi:10.3390/metabo11010023)
Supplement: Supplementary file 1 [file metabolites-11-00023-s001.pdf]

Supplementary Material 1.

Calculations of Free and Bioavailable 25-hydroxyvitamin D based on Vermeulen's equations:

Free 25(OH) vitamin D = Free [D]:

Free [D] = $\frac{-b+\sqrt{b^2-4ac}}{2a}$

Bioavailable (non-DBP bound vitamin):

Bio [D] = Free [D] + [DAlb] = (Kalb · [Alb] + 1) · Free [D]

EXAMPLE CALCULATION

Total 25(OH)-vitamin D = [Total] = 40 ng/mL = 1.0×10^{-7} mol/L

Total serum VDBP = [Total VDBP] = 250 ug/mL = 4.3×10^{-6} mol/L

Total serum albumin = [Alb] = 4.3 g/dL = 6.4×10^{-4} mol/L

K_{alb} = *affinity constant between 25(OH)D and albumin = 6 x* 10^{5} M^{-1}

K_{VDBP} = *affinity constant between 25(OH)D and VDBP = 7 x* 10^{8} M^{-1}

a = 2.7×10^{11}

b = 3325

c = $- 1 \times 10^{-7}$

Calculated concentration of free 25(OH)D = 3.01×10^{-11} mol/L = 12.1 pg/mL

Calculated concentration of bioavailable 25(OH)D = 1.09×10^{-8} mol/L = 4.6 ng/mL

Then, values were substituted based on the conversion units from Powe's paper:

Example:

Total 25(OH) vitamin D = [Total 25OHD] = 66.00 nmol/L = convert to mol/L = [Total] nmol/L x 10^{-9} = 66.00 x 10^{-9} = **6.6 x 10^{-8} mol/L**

Total serum VDBP = [Total VDBP] = 272.5 ug/mL = convert to mol/L = [Total DBP] ug/mL x 1.72×10^{-8} = **4.6 x 10^{-6} mol/L**

Total serum albumin = [Alb] = 50.00 g/L = convert to mol/L = [Alb] g/L x 1.4884 x 10^{-5} = **7.4 x 10^{-4} mol/L**

K_{alb} = 6×10^{5} M^{-1}

$K_{VDBP} = 7.0 \times 10^{8}\ M^{-1}$

$a = 3.133 \times 10^{11}$

$b = 3682.22$

$c = -6.6 \times 10^{-8}$

a = KVDBP · Kalb · [Alb] + KVDBP

$= 7.0 \times 10^{8} \times 6 \times 10^{5} \times 7.4 \times 10^{-4}$ mol/L $+ 7.0 \times 10^{8}$

$= 3.133 \times 10^{11}$

b = KVDBP · [Total VDBP] – KVDBP · [Total 25OHD] + Kalb · [Alb] +1

$= [7.0 \times 10^{8} (4.6 \times 10^{-6}$ mol/L)] – [$7.0 \times 10^{8} \times 6.6 \times 10^{-8}$ mol/L]
+ [($6 \times 10^{5} \times 7.4 \times 10^{-4}$ mol/L) +1]

= 3280.9 – 46.2 + 447.5

= 3682.22

$$\text{Free [D]} = \frac{-b+\sqrt{b^2-4ac}}{2a}$$

$$= \frac{-3682.2 + \sqrt{(-3682.2)^2 - (4 \times 3.133 \times 10^{11} \times [(-6.6 \times 10^{-8})]}}{(2 \times 3.133 \times 10^{11})}$$

$$= \frac{-3682.2 + \sqrt{13{,}558{,}596.8 + 82{,}701.7}}{6.266 \times 10^{11}}$$

$$= \frac{-3682.2 + 3693.4}{6.266 \times 10^{11}}$$

$$= \frac{11.2}{6.266 \times 10^{11}} = 1.78742 \times 10^{-11} \text{ mol/L}$$

= convert to pmol/L

(*Please refer to Table S1 to convert Free 25(OH) D from mol/L to pmol/L*)

= Free [D] $\times 10^{12} = 1.78742 \times 10^{-11}$ mol/L $\times 10^{12}$ **= 17.897pmol/L**

Bio [D] = [D] + [DAlb] = (Kalb · [Alb] + 1) · [D]

$= [(6 \times 10^{5} \times 7.4 \times 10^{-4}$ mol/L) + 1] $\times 1.\ 78742 \times 10^{-11}$mol/L

$= 447.5 \times 1.\ 78742 \times 10^{-11}$

$= 8.0 \times 10^{-9}$ mol/L

= convert to nmol/L

(*Please refer to Table S1 to convert Bioavailable 25(OH)D from mol/L to nmol/L*)

= Bio [D] $\times 10^{9} = 8.0$ X 10^{-9} mol/L $\times 10^{9}$ = **8.0 nmol/L**

Footnotes:

1. ^The formula used to calculate bioavailable and free 25(OH)D concentration is based on Vermeulen method [64] and a study by Powe et al. [21].
2. Table S1 Conversion table structure is based on a study by Thambiah et al. [33].

Column	Variable	Unit conversion	Calculation	Vermeulen supplementary material
E	Albumin (B)	g/L → mol/L	$\frac{\text{value (B)} \times 6.4 \times 10^{-4}}{43}$ value (B) X 1.4884 X 10^{-5}	4.3g/dL = 6.4 X 10^{-4}mol/L 43g/L = 6.4 X 10^{-4}mol/L
F	25OHD (C)	nmol/L → mol/L	value (C) X 10^{-9}	
G	VDBP (D)	μg/mL → mol/L	$\frac{\text{value (D)} \times 4.3 \times 10^{-6}}{250}$ value (D) X 1.72 X 10^{-8}	250μg/mL = 4.3X10^{-6} mol/L
H	KVDBP	-	-	7.0 x 10^{8} M^{-1}
I	Kalb	-	-	6 x 10^{5} M^{-1}
J	a	-	H * I * E + H	KVDBP · Kalb · [Alb] + KVDBP
K	b	-	H * G – H * F + I * E + 1	KVDBP·[Total VDBP]–KVDBP ·[Total] +Kalb·[Alb] +1
L	c	-	- (value F)	-[Total Vit D]

Column	Variable	Unit conversion	Calculation	Vermeulen supplementary material
M	Free Vit D	-	(-K + SQRT (K * K – 4 * J * L)) / (2*J)	$\frac{-b+\sqrt{b^2-4ac}}{2a}$
N	Bio Vit D	-	(I * E + 1) * M	(Kalb ·[Alb] + 1) ·[D]
O	Free Vit D	mol/L → pmol/L	value (M) X 10^{12}	-
P	Bio Vit D	mol/L → nmol/L	value (N) X 10^{9}	-

FREE & BIOAVAILABLE VITAMIN D CALCULATIONS

Table S1. Conversion table for free and bioavailable 25(OH)D calculations
